# PREDAC-FluB: predicting antigenic clusters of seasonal influenza B viruses with protein language model embedding based convolutional neural network

**DOI:** 10.1093/bib/bbaf308

**Published:** 2025-07-16

**Authors:** Wenping Xie, Jingze Liu, Chuan Wang, Jiangyuan Wang, Wenjie Han, Yousong Peng, Xiangjun Du, Jing Meng, Kang Ning, Taijiao Jiang

**Affiliations:** College of Life Science and Technology, Huazhong University of Science and Technology, No. 1037 Luoyu Road, Wuhan 430074, Hubei Province, China; Guangzhou National Laboratory, No. 9 XingDaoHuanBei Road, Guangzhou International Bio Island, Guangzhou 510005, Guangdong Province, China; State Key Laboratory of Common Mechanism Research for Major Diseases, Suzhou Institute of Systems Medicine, Chinese Academy of Medical Sciences & Peking Union Medical College, No. 100 ChongWen Road, Suzhou 215123, Jiangsu Province, China; Ruogu Intelligent Technology (Suqian) Company, Limited, No. 110 Jiangshan Avenue, Suqian High-Tech Industrial Development Zone, Suqian 223801, Jiangsu Province, China; Guangzhou National Laboratory, No. 9 XingDaoHuanBei Road, Guangzhou International Bio Island, Guangzhou 510005, Guangdong Province, China; Ruogu Intelligent Technology (Suqian) Company, Limited, No. 110 Jiangshan Avenue, Suqian High-Tech Industrial Development Zone, Suqian 223801, Jiangsu Province, China; College of Life Science and Technology, Sun Yat-sen University, No. 135 Xingang Xi Road, Guangzhou, 510275, Guangdong Province, China; Guangzhou National Laboratory, No. 9 XingDaoHuanBei Road, Guangzhou International Bio Island, Guangzhou 510005, Guangdong Province, China; School of Public Health (Shenzhen), Sun Yat-sen University, No. 135 Xingang Xi Road, Guangzhou, 510275, Guangdong Province, China; College of Biology, Hunan University, Lushan Road (S), Yuelu District, Changsha 410082, Hunan Province, China; School of Public Health (Shenzhen), Sun Yat-sen University, No. 135 Xingang Xi Road, Guangzhou, 510275, Guangdong Province, China; Ruogu Intelligent Technology (Suqian) Company, Limited, No. 110 Jiangshan Avenue, Suqian High-Tech Industrial Development Zone, Suqian 223801, Jiangsu Province, China; School of Information Engineering, Suqian University, No. 399 HuangheNan Road, Suqian 223800, Jiangsu Province, China; College of Life Science and Technology, Huazhong University of Science and Technology, No. 1037 Luoyu Road, Wuhan 430074, Hubei Province, China; College of Life Science and Technology, Huazhong University of Science and Technology, No. 1037 Luoyu Road, Wuhan 430074, Hubei Province, China; Guangzhou National Laboratory, No. 9 XingDaoHuanBei Road, Guangzhou International Bio Island, Guangzhou 510005, Guangdong Province, China

**Keywords:** seasonal influenza B viruses, HA sequence, antigenic variant, antigenic cluster, convolutional neural network

## Abstract

Influenza poses a significant global public health threat, with vaccination being the most effective and economical preventive measure. However, these punctuated antigenic changes, particularly in HA, result in escape from the immunity that was induced by prior infection or vaccination. Accurately predicting antigenic variation and understanding the antigenic dynamics of influenza viruses are crucial for selecting appropriate vaccine strains, but no established methods exist for influenza B viruses. Therefore, we present PREDAC-FluB, a hybrid deep learning framework that integrates spatial feature extraction via CNN to model interactions in HA1 sequences, multimodal sequence representation combining ESM-2 embeddings with six physicochemical descriptors and continuous encoding (ESM2–7-features), and UMAP-guided clustering for antigenic cluster identification. Using data from 9036 B/Victoria-lineage and 4520 B/Yamagata-lineage influenza virus pair. PREDAC-FluB demonstrates superior performance over traditional machine learning methods in predicting antigenic variation in influenza viruses, successfully identifying major antigenic clusters. Specifically, PREDAC-FluB classified the B/Victoria lineage into nine antigenic clusters and the B/Yamagata lineage into three antigenic clusters. In five-fold cross-validation for B/Victoria viruses, PREDAC-FluB with ESM2–7-features encoding achieved AUROC values of 0.9961 on the validation set and 0.9856 on the independent test set. In retrospective testing for B/Victoria viruses, PREDAC-FluB achieved AUROC values ranging from 0.83 to 0.97, demonstrating high prediction accuracy and effectively capturing antigenic variation information. In conclusion, PREDAC-FluB is a robust tool for antigenic computation, capable of accurately predicting antigenic variation in influenza B viruses. Its high prediction accuracy makes it a promising auxiliary method for recommending future influenza vaccine strains.

## Introduction

Seasonal influenza viruses represent a long-term threat to human health, causing significant morbidity and mortality annually worldwide [1]. Vaccination stands as the most effective and cost-efficient way for prevention and management of influenza, with the surface antigen hemagglutinin (HA) serving as the main constituent of influenza vaccines [2]. However, these punctuated antigenic changes, particularly in HA, result in escape from the immunity that was induced by prior infection or vaccination, thus allowing the virus to reinfect individuals who were once immune to the virus and necessitating reformulation of the seasonal influenza virus vaccine [3]. This challenges global vaccine recommendation efforts and vaccine effectiveness [4–6]. Therefore, accurately predicting antigenic variants and understanding the antigenic dynamics of influenza viruses are crucial for effective vaccine recommendations.

Seasonal influenza viruses, including types A and B, both circulate among humans. Influenza A viruses have subtypes A/H1N1 and A/H3N2, while influenza B viruses have two lineages: B/Victoria and B/Yamagata. These lineages are antigenically and genetically distinct, and since March 2020, all classified influenza B viruses belong to the B/Victoria lineage [7]. Compared with influenza A viruses, seasonal influenza B viruses cause fewer and less severe outbreaks, and have been explored to a smaller degree [3], and thus have been the subject of relatively fewer studies.

Traditional characterization of antigenic variants relies on labor-intensive and time-consuming hemagglutination inhibition (HI) assays [8]. To address these limitations, computational approaches have been developed. For instance, Smith et al. utilized metric multidimensional scaling to construct an antigenic map for influenza A/H3N2 viruses, revealing their clustered evolutionary patterns [9]. Subsequent studies have focused on developing computational models to predict antigenic evolution, primarily for influenza A viruses [10–17]. Notably, influenza B viruses are still responsible for ~25% of global influenza hospitalization cases [18], and their antigenic evolution remains poorly understood compared to influenza A. This disparity stems from two key challenges: This disparity stems from two key challenges: (i) the lower mutation rate of influenza B HA proteins, leading to subtle antigenic drift patterns that traditional models struggle to capture; and (ii) the scarcity of surveillance data for influenza B viruses, particularly for certain lineages, which hinders comprehensive understanding of their antigenic evolution. Additionally, the assessment of vaccine cross-protection is also challenged by the lack of sufficient surveillance data.

In our previous work, we developed PREDAC-CNN, a method that has been instrumental in predicting the antigenic evolution of seasonal influenza A viruses, as evidenced by its application in the seasonal influenza. This method leverages a convolutional neural network (CNN) model to capture interactions between amino acid sites in the HA1 sequence and to analyze the collective impact of individual mutations on antigenic variation [17]. Building on the strengths of PREDAC-CNN, we further extended this framework to develop PREDAC-FluB (Predicting Antigenic Clusters of influenza B viruses), which is designed to predict the antigenic evolution of seasonal influenza B viruses. PREDAC-FluB incorporates the six physicochemical features included in PREDAC-CNN. These features are crucial for characterizing the physicochemical properties of key antigenic proteins, such as HA. However, physicochemical features, which are predominantly local statistics, can reflect the properties of amino acids but fail to capture long-range co-evolutionary patterns or dependencies between functional sites within the sequence. This may lead to insufficient characterization of complex antigenic variations, especially in influenza B viruses, which have a lower mutation rate. As a result, it may be challenging to effectively capture complex antigenic evolutionary patterns and may lack sufficient generalization ability when confronted with new antigenic variants. In light of these limitations, we introduced embeddings from the pre-trained protein language model ESM-2 (Evolutionary Scale Modeling version 2) to capture global sequence patterns and evolutionary information. ESM-2 is capable of encoding the ‘semantic meaning’ of each amino acid in the HA1 sequence [19], thereby complementing the limitations of physicochemical features. Numerous studies have shown that combining ESM-2 embeddings with physicochemical features significantly outperforms using either feature set alone [20, 21].

To further explore the antigenic diversity within the B/Victoria and B/Yamagata lineages, we used an assisted clustering strategy based on uniform manifold approximation and projection (UMAP) to divide antigenic clusters [22, 23], which provides more accurate and interpretable results than traditional clustering methods (such as K-means). The PREDAC-FluB model, developed in this study, integrates convolutional neural networks, ESM-2 language model embeddings, and amino acid physicochemical characteristics to provide an efficient tool for predicting the antigenic evolution of influenza B virus. The model accurately identifies antigen clusters, offering crucial insights for the selection of vaccine strains and effective monitoring of antigens, as evidenced by the methodologies and strategies detailed in recent research on vaccine development. In addition, we show how antigen cluster dynamics can be used to predict vaccine formulation adjustments, revealing the impact of antigen drift on vaccine efficacy. Nevertheless, the antigenic evolution of influenza viruses is also influenced by complex factors such as immunoimprinting and population immunity, which provides a new direction for future research. This study offers a significant contribution to the field by proposing a novel vaccine strategy that targets both the Victoria and Yamagata lineages of influenza B virus, potentially enhancing cross-protection and advancing the development of more effective vaccines.

## Materials and methods

### Datasets

HI measurements for influenza B/Victoria and B/Yamagata lineages were collected from reports of the Worldwide Influenza Centre lab to the World Health Organization (WHO) (https://www.crick.ac.uk/research/platforms-and-facilities/worldwide-influenza-centre/annual-and-interim-reports). HA sequences were collected from the Global Initiative on Sharing All Influenza Data (GISAID) [24]. To ensure data quality, redundant HA1 sequences were removed using a 100% sequence identity threshold. Strain pairs exceeding 15 amino acid substitutions in HA1 were excluded through a three-step filtering pipeline [12]: (i) sequence alignment with MAFFT v7.505, (ii) pairwise substitution calculation using Biopython’s pairwise2 module, and *(iii)* elimination of pairs violating the substitution limit. This yielded 9036 B/Victoria strain pairs (2001–2023) and 4520 B/Yamagata strain pairs (1994–2020) for model development.

For antigenic cluster inference, HA1 sequences spanning 1974–2024 were retrieved from GISAID [24]. Initial redundancy reduction by 100% sequence identity produced 6249 B/Victoria and 2677 B/Yamagata unique sequences. To further refine B/Victoria lineage data, CD-HIT [25] clustering was applied, retaining 3439 representative sequences. Phylogenetic trees were reconstructed using FastTree v2.1.11 [26], enabling lineage-specific evolutionary analysis.

### Definition of antigenic relationships

Antigenic distance ${D}_{ab}$ between paired strains is represented as follows references:


(1) \begin{equation*} {D}_{ab}=\frac{H_{aa}}{H_{ab}} \end{equation*}

where ${H}_{ab}$ indicates the HI titer of strain a necessary to inhibit cell agglutination induced by strain $\mathrm{b}$. Two strains are deemed antigenic variants if their antigenic distance is 4 or more. If the distance is less, they are deemed antigenically similar [27, 28]. As a result, for the 9036 strain pairs of influenza B/Victoria lineage, 7061 pairs were classified as antigenically distinct (positive) and 1975 pairs as antigenically similar (negative). For the 4520 strain pairs of influenza B/Yamagata lineage, 2207 pairs were identified as having distinct antigenic properties (positive), indicating significant differences in their surface proteins, while 2313 pairs were classified as antigenically similar (negative), suggesting minor or no differences in their HA and neuraminidase proteins. The distributions of antigenically distinct and similar strain pairs for influenza B/Victoria and B/Yamagata lineages are detailed in Tables 1 and 2, respectively, reflecting the ongoing antigenicity evolution.

**Table 1:** The distributions of antigenically distinct strain pairs and antigenically similar strain pairs for each year for influenza B/Victoria viruses.

**Year**	**Antigenically similar**	**Antigenically distinct**	**Year**	**Antigenically similar**	**Antigenically distinct**
2002	1	0	2014	67	221
2004	2	0	2015	90	184
2005	1	0	2016	117	654
2006	0	1	2017	102	528
2007	14	9	2018	83	626
2008	61	41	2019	177	1294
2009	8	14	2020	97	681
2010	94	165	2021	165	481
2011	108	184	2022	173	790
2012	240	445	2023	230	526
2013	145	217			

*Note.* For each specific year, a strain pair contains a strain isolated on that year and the other strain isolated on or before that year.

**Table 2:** The distributions of antigenically distinct strain pairs and antigenically similar strain pairs for each year for influenza B/Yamagata viruses.

**Year**	**Antigenically similar**	**Antigenically distinct**	**Year**	**Antigenically similar**	**Antigenically distinct**
1998	1	0	2013	285	338
2006	1	0	2014	235	259
2007	10	8	2015	315	237
2008	24	21	2016	128	216
2009	10	0	2017	410	454
2010	55	23	2018	488	294
2011	61	58	2019	64	71
2012	217	226	2020	9	2

*Note.* For each specific year, a strain pair contains a strain isolated on that year and the other strain isolated on or before that year.

### UMAP-assisted K-means clustering for antigenic characterization

This section details the methodology employed for antigenic clustering, combining UMAP for.

dimensionality reduction and K-means for cluster assignment. The process can be broken down into the following steps:

1. Representation of Influenza Samples and Antigenic Distance:

Let *S* = {s_1_, s_2_, …, s_n_} represent a set of *n* influenza virus samples. The antigenic relationships between these samples are captured in an antigenic correlation matrix *A* ∈ ℝ^n × n^, where each element *a*_ij_ represents the antigenic similarity between samples *s*_i_ and *s*_j_. This similarity can be derived from experimental data, such as HI assays, or predicted using computational models like PREDAC-FluB. The antigenic distance d_ij_ between samples s_i_ and s_j_ can be defined as a measure of the extent of antigenic variation between two viral strains, which is crucial in understanding the evolution and spread of influenza viruses:


(2) \begin{equation*} {d}_{ij}=1-{a}_{ij} \end{equation*}

This distance metric ranges from 0 (identical antigenicity) to 1 (maximum antigenic dissimilarity).

2. Dimensionality Reduction with UMAP:

UMAP is employed to reduce the dimensionality of the antigenic distance matrix while preserving the global structure of the data. UMAP constructs a high-dimensional graph representing the relationships between samples based on their antigenic distances.

(a) **Finding Nearest Neighbors:**
  For each sample *s*_i,_ its *k*-nearest neighbors are identified based on the antigenic distance *d*_ij_. Let *N*_i_ = {s_i1_, s_i2_, …, s_ik_} be the set of *k*-nearest neighbors of *s*_i_.
(b) **Computing Local Connectivity and Distances:**
  UMAP computes a local connectivity measure *ρ*_i_ for each sample *s*_i_, representing the distance to its closest neighbor:
(3) \begin{equation*} {\rho}_i={min}\left\{{d}_{ij}|{s}_j\in{N}_i,{d}_{ij}>0\right\} \end{equation*}
  Then, for each edge connecting *s*_i_ to its neighbor *s*_j_ ∈ *N*_i_, a weight *w*_ij_ is assigned based on the distance *d*_ij_ and the local connectivity *ρ*_i_:
  (4) \begin{equation*} {w}_{ij}={exp}\left(-\frac{{max}\left(0,{d}_{ij}-{\rho}_i\right)}{\sigma_i}\right) \end{equation*}
  where *σ*i is a scaling parameter that controls the width of the exponential kernel.
(c) **Constructing the Low-Dimensional Embedding:**
  UMAP then constructs a low-dimensional representation *Y* ∈ ℝ^n × m^ (where *m* is the desired dimensionality, typically 2 or 3) that preserves the topological structure of the high-dimensional graph. This is achieved by minimizing a cross-entropy loss function that compares the pairwise similarities in the high-dimensional space (represented by *w*_ij_) with the pairwise similarities in the low-dimensional space (represented by a function of the Euclidean distance between the lowdimensional coordinates *y*_i_ and *y*_j_). The optimization process involves finding the coordinates *y*_i_ that minimize the following loss function:
  (5) \begin{equation*} L=\sum_{i\ne j}\left[{w}_{ij}\log \left(\frac{w_{ij}}{p_{ij}}\right)+\left(1-{w}_{ij}\right)\log \left(\frac{1-{w}_{ij}}{1-{p}_{ij}}\right)\right] \end{equation*}
  where *p*_ij_ is the similarity between *y*_i_ and *y*_j_ in the low-dimensional space, typically defined as:
  (6) \begin{equation*} {p}_{ij}={\left(1+a{\left({y}_i-{y}_j\right)}^{2b}\right)}^{-1} \end{equation*}

In the context of similarity functions, hyperparameters such as a and b are crucial as they control the shape and behavior of the function, influencing how the similarity between two entities is computed.

3. K-means Clustering:

Following dimensionality reduction, the K-means algorithm is applied to the low-dimensional representation *Y* to assign samples to antigenic clusters. Let *C* = {c_1_, c_2_, …, c_k_} be the set of *K* cluster centroids. K-means aims to minimize the within-cluster sum of squares (WCSS):


(7) \begin{equation*} WCSS=\sum_{k=1}^K\sum_{y_{i\in{c}_k}}{\left\Vert{y}_i-{\mu}_k\right\Vert}^2 \end{equation*}

where *μ*_k_ is the centroid of cluster *c*_k_, calculated as the mean of the coordinates of all samples assigned to that cluster:


(8) \begin{equation*} {\mu}_k=\frac{1}{\left|{c}_k\right|}\sum_{y_{i\in{c}_k}}{y}_i \end{equation*}

The K-means algorithm is an iterative process that assigns samples to the nearest cluster centroid and updates the centroids based on the average of the samples within each cluster. The centroid positions until convergence.

4. Antigenic Cluster Assignment:

After the K-means algorithm converges, each sample s_i_ is assigned to an antigenic cluster c_k_ based on its low-dimensional representation y_i_. The resulting clusters represent groups of samples with similar antigenic properties. This UMAP-assisted K-means clustering approach provides a robust and efficient method for identifying antigenic clusters in influenza viruses, enabling a deeper understanding of their antigenic evolution and informing public health interventions.

### Calculating the antigenicity coverage of the vaccine strain

To calculate the antigenicity coverage of WHO-recommended vaccine strains, 6249 and 2677 non-redundant HA1 sequences were retained as representative proteins for further analysis for Victoria lineage and Yamagata lineage, respectively. And we used the PREDAC-FluB frame to predict antigenic relationships between pairwise strains.

The antigenicity coverage of each WHO-recommended vaccine strain in each year was defined as follows [29]:


(9) \begin{equation*} {VC}_{YN}=\frac{C}{M} \end{equation*}

where ${VC}_{YN}$ means the vaccine coverage for year N, M means the total number of emerging strains collected in year N, C means the number of antigenic similar strains of the vaccine strain in M.

### Experimental design

To evaluate the performance of PREDAC-FluB in predicting antigenic relationships of seasonal influenza B viruses, we conducted two types of benchmarking experiments: 5-fold cross-validations and retrospective tests. These experiments assessed how different feature encodings impact PREDAC-FluB’s performance. For comparison, we included three state-of-the-art (SOTA) feature encodings: AAindex-PCA [15], ESM-2 [19], and iFeatureOmega [30] and evaluated four machine learning algorithms: logistic regression (LR), K-nearest neighbor (KNN), neural network (NN), and support vector machine (SVM). For each feature encoding method, we performed a comparative analysis between PREDAC-FluB and these four algorithms. For 5-fold cross-validations, we divided the dataset into training, validation, and test sets to calibrate, tune, and assess the model, respectively. For retrospective tests, we focused on the real-world scenario of vaccine recommendation, where vaccine effectiveness depends on the antigenic similarity between the vaccine strain and circulating strains during a specific influenza season [31]. A key task is to determine the antigenic relationship between the vaccine strain and strains expected to circulate in the upcoming season.

In conducting retrospective tests, we considered the isolation dates of each strain pair, with a particular focus on the Victoria and Yamagata lineages, given the importance of the Victoria and Yamagata lineages in WHO-recommended vaccines, our tests focused on strain pairs isolated from 2014 to 2023 for B/Victoria lineage viruses and from 2014 to 2020 for B/Yamagata lineage viruses, due to limited data before 2013. For B/Victoria lineage viruses, models were trained on data from 2001 to the year preceding the target year and tested on data from the target year. The same approach was used for B/Yamagata lineage viruses. For example, for B/Victoria lineage strains in 2014, the model was trained on data from 2001 to 2013 and tested on pairs of strains, one isolated in 2014 and the other on or before that year, as detailed in influenza surveillance reports.

To evaluate the performance of the models in predicting antigenic relationships, we used two key statistical metrics: precision and recall. These metrics are defined as follows:


(10) \begin{equation*} Precision=\frac{TP}{TP+ FP} \end{equation*}

(11) \begin{equation*} Recall=\frac{TP}{TP+ FN} \end{equation*}

where TP (True Positive) denotes the number of strain pairs correctly predicted to be antigenically distinct. FP (False Positive) denotes the strain pairs incorrectly predicted to be antigenically distinct. TN (True Negative) denotes the strain pairs correctly predicted to be antigenically similar. FN (False Negative) denotes the strain pairs incorrectly predicted to be antigenically similar. The F_1_ score is the harmonic mean of precision and recall, and indicates the models’ overall performance. Its formula is defined as follows:


(12) \begin{equation*} {F}_1=\frac{2\times precision\times recall}{precision+ recall} \end{equation*}

## Results

### Overview of PREDAC-FluB

**Figure 1:**
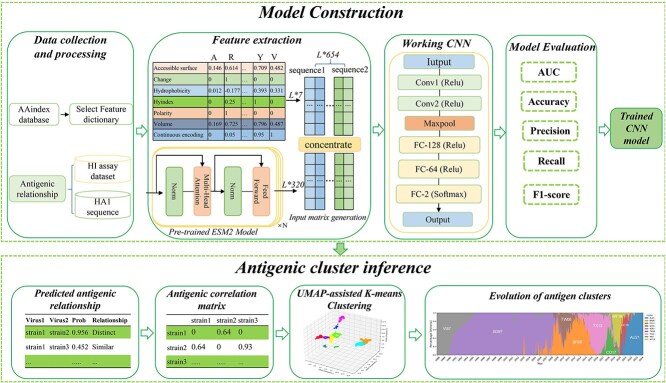
The workflow of PREDAC-FluB. PREDAC-FluB takes paired HA1 sequences of influenza B viruses from the B/Victoria lineage or B/Yamagata lineage as input. It generates input matrices corresponding to the paired HA1 sequences (sequence 1 and sequence 2) by encoding the selected physicochemical features of amino acids using a feature dictionary and obtaining embeddings from the pre-trained protein language model ESM-2 in the context of the HA1 sequences. Subsequently, PREDAC-FluB evaluates the input matrices using a trained CNN model to predict the antigenic relationships between paired strains of the B/Victoria lineage or B/Yamagata lineage. And it constructs an antigenic relatedness matrix and infers antigenic clusters using a UMAP-assisted K-means clustering method.

In this study, we developed PREDAC-FluB (Fig. 1), a Convolutional Neural Network (CNN)-based tool for predicting antigenic evolution in seasonal influenza B viruses. PREDAC-FluB processes paired HA1 sequences from the B/Victoria and B/Yamagata lineages and integrates six amino acid physicochemical properties, a continuous amino acid encoding, and ESM-2 embeddings (7-dimensional and 320-dimensional features, respectively) into a unified ‘ESM2–7-features’ encoding matrix Using this comprehensive input matrix, PREDAC-FluB trains the CNN model and selects the optimal model. Subsequently, the tool employs the trained model to predict the antigenic relationships between paired influenza B viruses and constructs an antigenic correlation matrix. Based on this matrix, PREDAC-FluB applies UMAP to project high-dimensional relationships into a 3D latent space, assigning spatial coordinates to each antigen. These coordinates are then partitioned into clusters using the K-means algorithm [32], enabling detailed antigenic clustering and evolutionary tracking. For more details about the structure of PREDAC-FluB, please refer to the Supplementary Materials.

### Performance evaluation of PREDAC-FluB

To evaluate the performance of different models in predicting antigenic relationships, we conducted benchmark experiments focusing on the impact of various feature encodings on CNN models and comparing CNNs to traditional machine learning algorithms. We tested four state-of-the-art (SOTA) feature encoding methods with four widely used machine learning algorithms. These encodings capture viral sequence antigenic properties, including sequence patterns, structural features, and evolutionary traits. In our study, we utilized ESM2, a deep learning-based protein language model, to enhance our computational protein optimization strategies. ESM2 captures deep sequence dependencies and extracts evolutionary information from large datasets, providing rich sequence representations. Additionally, we combined ESM2 embeddings with seven physicochemical feature encodings (ESM2–7-features) to leverage both deep learning and physicochemical properties, aiming to improve prediction accuracy.

**Figure 2:**
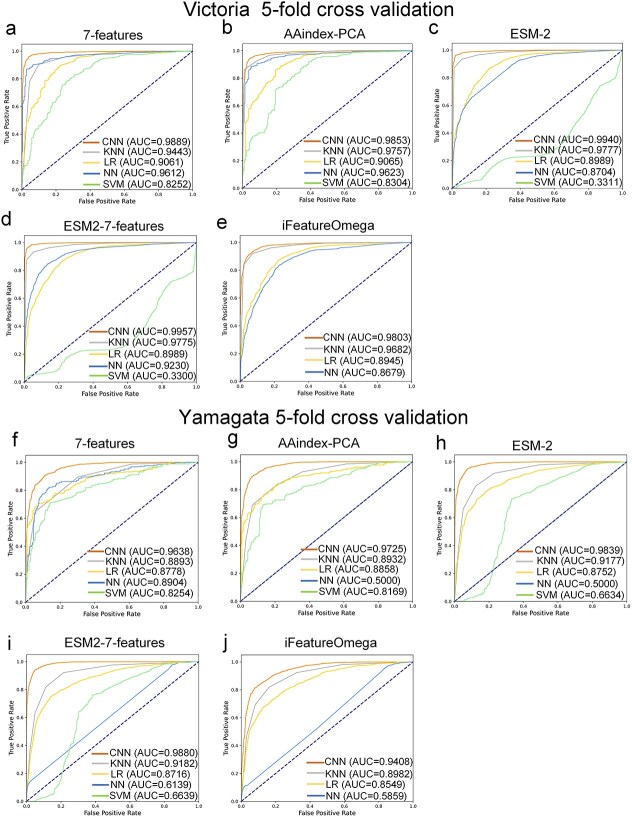
Performance of the CNN model was compared with four other machine learning models—Logistic regression (LR), K-nearest neighbors (KNN), neural networks (NN), and support vector machine (SVM) in the 5-fold cross-validation. These models were evaluated using five distinct feature encodings on validation datasets. For the influenza B/Victoria lineage, the feature encodings are as follows: 7-features (**a**), AAindex-PCA (**b**), ESM-2 embeddings (**c**), combined ESM2–7-features (**d**), and iFeatureOmega (**e**). Correspondingly, for the influenza B/Yamagata lineage, the feature encodings are presented in panels (**f**) through (**j**): 7-features (**f**), AAindex-PCA (**g**), ESM-2 embeddings (**h**), ESM2–7-features (**i**), and iFeatureOmega (**j**).

**Figure 3:**
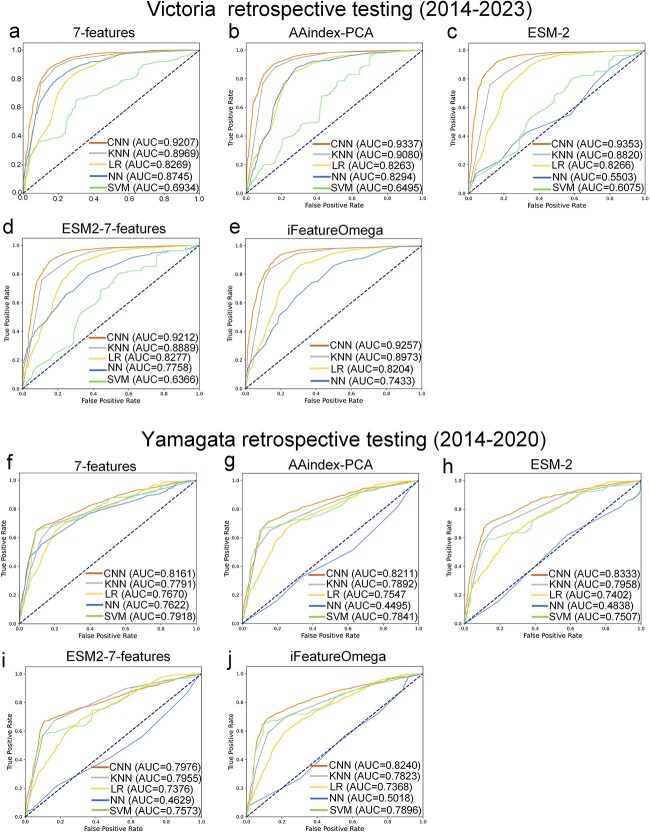
The performance of the convolutional neural network (CNN) was compared with four other machine learning models—Logistic regression (LR), K-nearest neighbors (KNN), neural networks (NN), and support vector machine (SVM)—Using five feature encodings in retrospective testing. ROC curves for feature encoding of 7-features (**a**), AAindex-PCA (**b**), ESM-2 embeddings (**c**), ESM2–7-features (**d**) and iFeatureOmega (**e**) on influenza B/Victoria viruses. ROC curves for feature encoding of 7-features (**f**), AAindex-PCA (**g**), ESM-2 embeddings (**h**), ESM2–7-features (**i**), and iFeatureOmega (**j**) on influenza B/Yamagata viruses.

**Figure 4:**
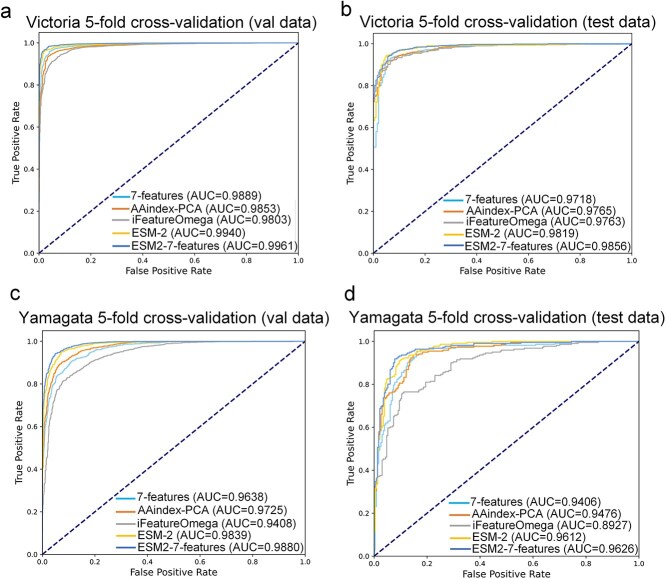
The performance of ‘ESM2–7-features’ feature encodings compared with other four different feature encodings with CNN model. ROC curves on the validation sets and the independent test sets for influenza B/Victoria viruses (**a-b**) and influenza B/Yamagata viruses (**c-d**).

For each feature encoding method, we compared the CNN model with four traditional machine learning models—Logistic Regression (LR), K-Nearest Neighbors (KNN), Neural Networks (NN), and Support Vector Machine (SVM) to assess their ability to predict antigenic relationships, sensitivity to sequence variations, and overall performance. The comparison allows us to pinpoint the most effective combination of feature encoding and model for predicting viral antigenic behavior. The results show that, among the five feature encodings evaluated, the CNN model outperformed the other four traditional machine learning models on the validation set (see Fig. 2a-2j). Specifically, for the B/Victoria lineage, the AUC values of the CNN model were: 7-features (0.9889), AAindex-PCA (0.9853), ESM-2 (0.9940), ESM2–7-features (0.9957), and iFeatureOmega (0.9803). For the B/Yamagata lineage, the AUC values were: 7-features (0.9638), AAindex-PCA (0.9725), ESM-2 (0.9839), ESM2–7-features (0.9880), and iFeatureOmega (0.9408). In retrospective testing, the CNN model demonstrated superior performance over four other models across 10 subsets of the B/Victoria lineage and seven subsets of the B/Yamagata lineage, as evidenced by Fig. 3a-3j. When the ESM2 embedding was combined with the physicochemical feature encoding of amino acids (ESM2–7-features) and applied to the CNN model, this feature encoding showed superior performance to the other four encodings in both the validation set and the independent test set of the B/Victoria and B/Yamagata lineages (see Fig. 4a-4d). This highlights the significant impact of the convolutional architecture of the CNN model on processing HA1 sequences, especially in capturing the complex interactions between amino acids and spatial orientation information. Therefore, by combining the evolutionary information of ESM2 with the physicochemical feature encoding of amino acids, the advantages of CNN in extracting sequence features can be fully utilized to reveal the overall impact of subtle changes in HA1 sequence on antigenic variation.

### Application of PREDAC-FluB to capture antigenic evolution of influenza B viruses

**Figure 5:**
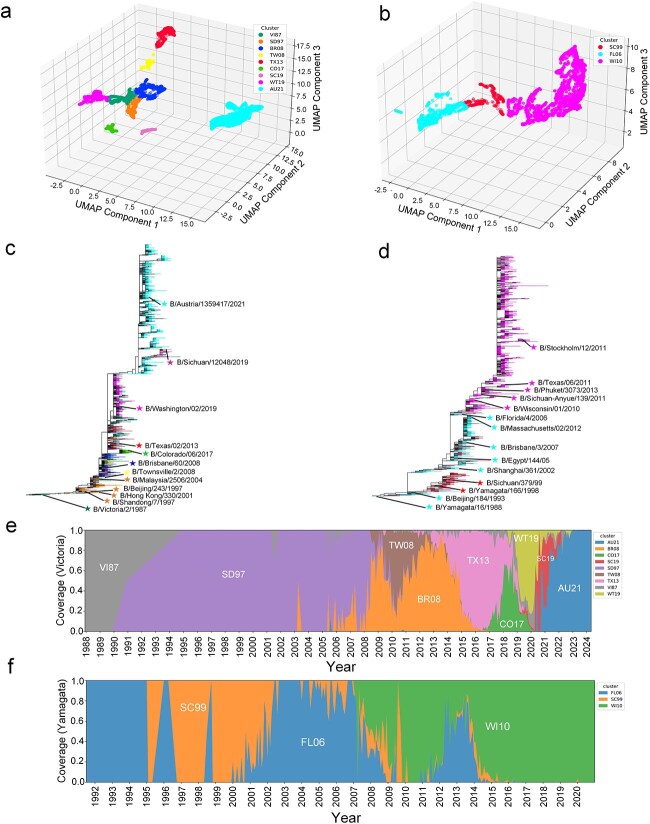
Antigenic clusters and genetic evolution of influenza B/Victoria viruses and influenza B/Yamagata viruses. (**a-b**) the spatial distribution of these clusters in a three-dimensional UMAP space. The antigenic clusters are identified by abbreviations corresponding to the earliest vaccine strains recommended by the WHO and strains that circulated during the respective flu seasons. For influenza B/Victoria viruses, these include B/Victoria/2/1987 (VI87), B/Shandong/7/1997 (SD97), B/Brisbane/60/2008 (BR08), B/Townsville/2/2008 (TW08), B/Texas/02/2013 (TX13), B/Colorado/06/2017 (CO17), and B/Austria/1359417/2021 (AU21). For influenza B/Yamagata viruses, the clusters are represented by B/Sichuan/379/99 (SC99), B/Florida/4/2006 (FL06), and B/Wisconsin/01/2010 (WI10). (**c-d**) the genetic evolution of influenza B/Victoria viruses and influenza B/Yamagata viruses, with WHO-recommended vaccine strains and circulated strains in the corresponding flu season denoted by five-pointed stars. (**e-f**) the percentage of each antigen cluster of influenza B/Victoria viruses and influenza B/Yamagata viruses in different influenza seasons.

To elucidate the antigenic diversity within the two major lineages of influenza B viruses, we employed a UMAP-assisted clustering approach to delineate antigenic clusters for both influenza B/Victoria (spanning 1987–2024) and B/Yamagata (spanning 1988–2024) viruses. This method effectively captures complex antigenic relationships and projects them into a lower-dimensional space, facilitating the identification of distinct antigenic groups. When determining the optimal number of clusters, we first examined the trend of the WCSS and identified the point where the WCSS began to level off (see Supplementary Fig. S3), which indicated the best fit for the data. Subsequently, we calculated the proportion of antigenically variant strains correctly assigned to different antigenic clusters based on the HI titration relationships among the circulating strains, using this as the initial basis for classification. On this foundation, we further refined the clustering of antigenic clusters by incorporating phylogenetic information from evolutionary trees (Fig. 5c and 5d). Ultimately, we identified nine major antigenic clusters for B/Victoria lineage and three antigenic clusters for B/Yamagata lineage (see Fig. 5a and 5b).

For B/Victoria viruses, Fig. 5a illustrates the antigenic clustering of influenza B viruses belonging to the Victoria lineage. Nine distinct antigenic clusters were identified, each represented by a unique color. The spatial separation of these clusters in the three-dimensional UMAP space signifies antigenic divergence. Notably, the clusters represented by cyan (AU21) and magenta (WT19) exhibit the greatest spatial separation, suggesting substantial antigenic differences between these groups. Conversely, clusters such as orange (SD97) and dark blue (VI87) are positioned in closer proximity, indicating a higher degree of antigenic similarity. The remaining clusters are distributed throughout the antigenic space, reflecting a spectrum of antigenic variation within the Victoria lineage. The analysis revealed that antigenic clusters dominating influenza B/Victoria strains persisted for an average of 4.6 years. Notably, only one cluster dominated in each season before 2001, but two or three clusters became prevalent thereafter. Bedford and colleagues categorized four main clusters: VI87, HK01, MA04, and BR08 from 1986 to 2011, and these antigenic clusters demonstrated a high degree of genetic similarity in their study. In our research, during the same period, three main clusters were formed: VI87, SD97, and BR08, which included strains used for vaccine development. Interestingly, the strains B/Shandong/7/97, B/Hong Kong/330/2001, and B/Malaysia/2506/2004 exhibited antigenic similarities, and were accurately classified into the SD97 cluster by the PREDAC-FluB model, aligning with Bedford et al.’s findings [33]. Furthermore, antigenic analysis shows that most recent viruses belong to the genetic subgroups 1A.3a.1 or 1A.3a.2, with the 1A.3a.2 subgroup being more common and having a wider geographical distribution [34]. Our study found that since the 2020 flu season, the SC19 cluster (1A.3a.1 subgroup) has been competing with the AU21 cluster (1A.3a.2 subgroup), with the AU21 cluster gradually becoming more prevalent (Fig. 5e). Ferret antisera raised against 1A.3 viruses, such as the 2021–2022 NH vaccine virus B/Washington/02/2019, struggle to recognize these emerging strains. Specifically, antisera against B/Sichuan-Jinyang/12048/2019 (1A.3a.1) better identify 1A.3a.1 viruses but poorly recognize 1A.3a.2 viruses. Conversely, antisera against B/Austria/1359417/2021 (1A.3a.2) better identify 1A.3a.2 viruses but not others. This indicates significant antigenic differences between the two virus strains [34]. PREDAC-FluB effectively captured these differences, classifying the strains into distinct antigenic clusters and demonstrating the shift in dominant clusters (Fig. 5a).

For influenza B/Yamagata viruses, Fig. 5b depicts the antigenic clustering of influenza B viruses from the Yamagata lineage. Three distinct antigenic clusters were identified. The magenta cluster (WI10) occupies a large portion of the antigenic space, suggesting that many strains within this lineage share a similar antigenic profile. The red cluster (SC99) and cyan cluster (FL06) are located on opposite sides of the magenta cluster, indicating antigenic divergence from the predominant group. Additionally, antigenic clusters that were previously dominant continue to persist at lower proportions in subsequent years. The spatial distribution of these clusters reveals the antigenic heterogeneity present within the Yamagata lineage, albeit to a lesser extent than observed in the Victoria lineage. Our analysis found that antigenic clusters generally remained dominant for an average of 7.5 years, indicating slower antigenic evolution compared to the Victoria lineage. In our study, all vaccine strains with similar antigenicity were consistently grouped into the same antigenic clusters. Conversely, vaccine strains with distinct antigenicity were typically placed in different clusters. Where was observed with B/Shanghai/361/2002, B/Florida/4/2006 and B/Massachusetts/02/2012, which were the WHO-recommended vaccine strains grouped in the antigenic cluster FL06.

As shown in Fig. 5e and 5f, while the antigenic evolution of influenza B viruses is a continuous process, our findings further verify that their antigenic evolution tends to follow a pattern of distinct, cluster-wise changes [9]. Specifically, these antigenic clusters are characterized by continuous replacement, where each cluster is eventually succeeded by another cluster composed of strains with different antigenic properties. By comparing the clustering patterns of the two lineages, we have revealed significant differences in antigenic diversity: the Victoria lineage have more distinct antigenic clusters and a broader distribution in antigenic space, indicating higher antigenic variation compared to the Yamagata lineage. The result aligns with previous studies [35–37] reporting more pronounced antigenic drift within the Victoria lineage. Vaccine recommendations are guided by the emergence and prevalence of new antigenic clusters. If a new cluster emerges in September and its prevalence exceeds that of the existing dominant cluster, it is predicted to become dominant in the upcoming season, necessitating a vaccine strain update. If the new cluster does not surpass the existing dominant cluster in prevalence, the current cluster is expected to maintain its dominance, and a vaccine strain update is not necessary. Based on this criterion, we recommended updating the vaccine strain for the 2023 influenza season and continuing with the same strain for the 2024 season. Figure 5e illustrates that, had the vaccine recommendation been made in August 2023, B/Austria/1359417/2021 would have been chosen due to its highest proportion at that point. This predicted vaccine strain is consistent with the strain recommended by the WHO, further validating the utility and accuracy of our method.

### Detection of antigenic drift for vaccine strains

To evaluate the predictive capacity of our model for antigenic escape, we analyzed historical WHO-recommended influenza vaccine strains. The WHO annually designates vaccine strains, which are usually the same for both hemispheres, to provide broad protection against circulating variants during the target season [38]. Traditional methods rely on HI assays to assess antigenic match, whereas our sequence-based approach enables high-throughput predictions. The theoretical antigenicity coverage for each WHO-recommended vaccine strain was calculated as the proportion of antigenically similar strains among all circulating strains in the corresponding years.

For the Victoria lineage (1997–2024), Fig. 6a shows that most vaccine strains had low coverage (<50%), except for B/Shandong/7/1997 and B/Austria/1359417/2021. The coverage rates generally followed an inverted-V distribution, with an ascending-maintaining-descending shape. Among the seven vaccine strains, four were recommended before their peak coverage year, while three were recommended during the peak year. Notably, B/Shandong/7/1997 reached its highest coverage (78%) in 1997 but was recommended in 2000 when coverage had already dropped sharply.

**Figure 6:**
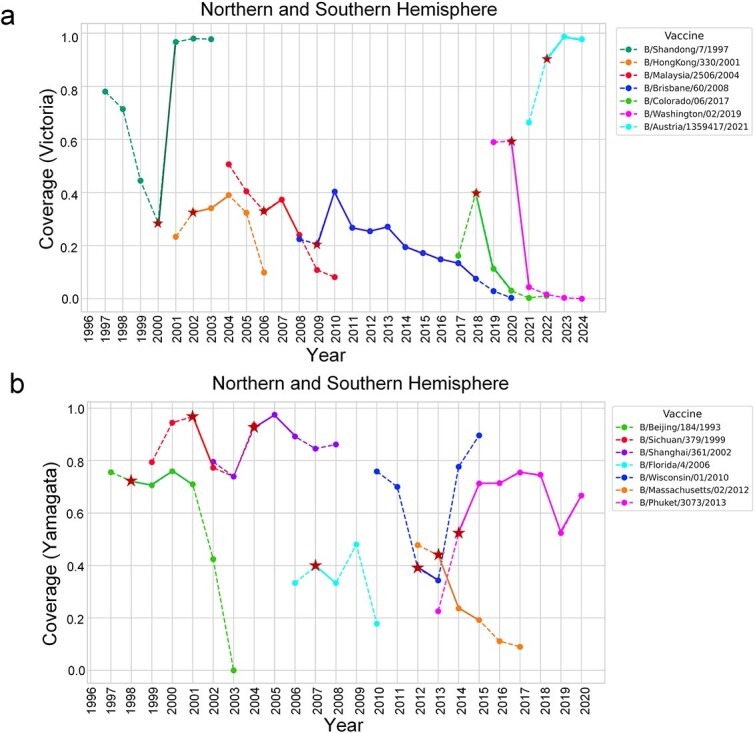
The coverage of with WHO-recommended vaccine in northern and southern hemisphere. (a-b) the x-axis represents the years from 1997 to 2024 for influenza B/Victoria viruses and from 1997 to 2020 for influenza B/Yamagata viruses. The y-axis indicates the vaccine coverage for each year. Each line depicts the antigenic coverage of a specific vaccine strain from its emergence year until two years after it is replaced by an updated vaccine strain. Stars denote the years when the vaccine strain was recommended. The solid line represents the influenza season in which the recommended vaccine strains were used, the dashed line indicates the time period from the initial emergence of the strain to its first recommendation as a vaccine strain, as well as the two years following its discontinuation.

For the Yamagata lineage (1997–2020), Fig. 6b shows that most vaccine strains provided broad protection against circulating strains within their recommended effective time. The coverage rates also followed an inverted-V distribution. For example, B/Sichuan/379/1999 and B/Shanghai/361/2002 had peak coverage at the time of recommendation, while B/Beijing/184/1993 and B/Massachusetts/02/2012 were recommended after their peak coverage year. B/Phuket/3073/2013 showed increased coverage post-recommendation and maintained this level, indicating sustained protection against future epidemic strains. Notably, B/Wisconsin/01/2010 maintained high coverage post-2013, indicating antigenic similarity with later strains.

Furthermore, the antigenic match between vaccine strains and circulating strains is crucial for vaccine efficacy. For example, during the 2023–2024 influenza season, the WHO recommended B/Austria/1359417/2021, which had over 95% coverage. The CDC reported vaccine effectiveness of 89% in children and 78% in adults (Supplementary Tables S5), indicating high protective efficacy when the vaccine strain is well-matched to circulating strains. However, antigenic drift can reduce vaccine effectiveness. However, antigenic drift can significantly reduce vaccine effectiveness. For example, in 2017, an antigenic change occurred in the HA evolutionary branch V1A.1 of the B/Victoria virus (with deletions of two amino acids at positions 162–163), and B/Colorado/06/2017 was selected as the reference virus for the 2018–2019 and 2019–2020 influenza seasons. However, starting in early 2019, B/Victoria viruses from the HA evolutionary branch V1A.3 (with deletions of three amino acids at positions 162–164) rapidly increased and almost completely replaced the V1A.1 viruses during the 2019–2020 influenza season. During this period, antigenic characterization showed reduced reactivity of B/Victoria viruses to ferret antisera raised against the vaccine components, indicating significant antigenic differences between the V1A.1 and V1A.3 subclades [39]. This finding aligns with the low coverage of B/Colorado/06/2017 during the 2019–2020 season (Fig. 6a), further validating our model’s sensitivity to antigenic drift.

According to the CDC, in the 2019–2020 influenza season, with B/Colorado/06/2017 as the reference virus for the B/Victoria lineage, the vaccine effectiveness against B/Victoria viruses was only 45% [4].

## Discussion

Antigenic drift and antigenic jump/shift in the HA surface antigen drive influenza virus immune escape and complicate the prediction of antigenic properties in emerging strains. However, accurate prediction of these variations is crucial for vaccine strain selection and maintaining vaccine efficacy. Traditional methods, relying on machine learning and statistical techniques, focus on specific amino acid sites (such as antigenic epitopes) [9, 40–44], lack scalability and fail to capture complex interactions among these sites, limiting prediction accuracy. To overcome these limitations, recent studies have shifted towards deep learning frameworks that simulate antigenic variation based on amino acid sequences and improve prediction accuracy [45]. For example, models like IAV-CNN [16] and PREDEAC-CNN [17] have integrated physicochemical features but primarily reflect local characteristics. The emergence of protein language models (such as ESM2), which capture evolutionary constraints and key features through pretraining, provides a more comprehensive understanding of viral evolution and mutation patterns. Combining evolutionary information with physicochemical properties [46] leverages the strengths of both approaches to enhance antigenicity prediction.

In this study, we developed the PREDAC-FluB model, which integrates evolutionary information from ESM-2 with the physicochemical encoding of amino acids to predict influenza B virus antigenicity. Our CNN model outperformed other machine learning models across five feature encoding methods, as shown in validation set results. On the independent test set, for Victoria viruses, the CNN model showed comparable performance to the KNN model when using ESM-2, ESM2–7-features, or iFeature-Omega encoding, but significantly outperformed other models with 7-features and AAindex-PCA encoding (see Fig. S1a-S1e). For Yamagata viruses, while the CNN model underperformed slightly than the KNN model when using iFeature-Omega encoding (AUC 0.8927 versus 0.9113), but outperformed others with alternative encodings (see Fig. S1f-S1j). This highlights the effectiveness of combining CNN with ESM2–7-features. Based on these results, we further evaluated the performance of CNN models combined with different feature encoding methods. Integrating ESM-2 evolutionary information with amino acid physicochemical properties (ESM2–7-features) enhances the model’s predictive capabilities for B/Victoria and B/Yamagata viruses. PREDAC-FluB achieved AUROC values of 0.9961 and 0.9856 for B/Victoria, and 0.9880 and 0.9626 for B/Yamagata in cross-validation and independent tests, respectively. This multimodal feature fusion approach provides a comprehensive solution for antigenicity prediction, overcoming single-feature limitations and improving performance and generalization.

Retrospectively, the optimal feature encoding, ESM2–7-features, exhibited marginally inferior performance compared to ESM-2 and AAindex-PCA feature encodings in the B/Victoria lineage, failing to surpass other encodings in the B/Yamagata lineage (Fig. S2a–2b). The result revealed that simply concatenating physicochemical properties with ESM-2 embeddings did not significantly improve the model’s generalization performance (see Fig. S2a) and, in some cases, even led to performance degradation (see Fig. S2b). Despite this, the results demonstrate that integrating ESM-2 enhances the model’s generalization ability, confirming the value of incorporating ESM-2 in the feature extraction module. This finding suggests that the suboptimal performance of the simple concatenation of these two features in retrospective testing does not imply their insignificance, but rather highlights the need for a more sophisticated fusion approach. By employing rational fusion strategies (e.g., shared latent space mapping), the complementary strengths of both modalities can be fully leveraged, allowing the model to enhance precision while maintaining generalization capability. In retrospective testing, for B/Victoria viruses, the AUROC values of PREDAC-FluB ranged from 0.83 to 0.97 (Supplementary Table S3). For B/Yamagata viruses, the AUROC values ranged from 0.78 to 1.0 (Supplementary Table S4). These discrepancies can be attributed to mutations in the HA sequence that were not accounted for during training, reflecting the inherent challenges posed by the evolutionary nature of influenza viruses. Overall, the robust performance of the model in cross-validation underscores its potential as a reliable tool for antigenicity prediction.

Influenza B viruses show complex evolutionary patterns, with the underlying factors still largely unknown. The HA gene in the Victoria and Yamagata lineages evolves slower than in influenza A. The Victoria lineage has a faster turnover rate (3.9–5.1 years) compared to the Yamagata lineage (6.3–7.2 years). The nucleotide substitution rates are 3.32 × 10^-3 per site per year for Yamagata and 1.32 × 10^-3 for Victoria [35–37]. Our study found average dominance periods of 4.6 years for B/Victoria and 7.5 years for B/Yamagata clusters, aligning with Smith et al.’s correlation between antigenic and genetic evolution. Our findings show that B/Yamagata viruses replace antigenic clusters less frequently than B/Victoria viruses. Notably, during the antigenic cluster analysis, a lineage named B/Townsville/2/2008, antigenically different from B/Brisbane/60/2008, continued until 2012 and formed a separate antigenic cluster. Previous studies have identified a minor lineage, B/Hubei-Songzi/51/2008, which antigenically distinct from B/Brisbane/60/2008, persisted until 2011 for Victoria lineage [47]. Although there is a lack of confirmatory experiments to prove whether B/Hubei-Songzi/51/2008 and B/Brisbane/60/2008 have an antigenic differences, a study showed that when dominant clusters from successive seasons belong to the same viral lineage, significant antigenic differences are always observed between viruses because, in this case, there must be direct selection to evade herd immunity [35]. Moreover, the Yamagata virus has not been detected since March 2020 in the B/Yamagata lineage of influenza viruses, a phenomenon that may be related to the non-pharmacological interventions implemented during the COVID-19 pandemic (such as social distancing, home isolation, etc.) that significantly reduced the spread of respiratory viruses [48, 49]. Furthermore, the antigenic characteristics of the virus itself are relevant; studies have indicated that the B/Yamagata lineage tends to exhibit slower antigenic drift, fewer amino acid changes and weak antigen selection [48], leading to reduced immune escape and a more stable immune response, thereby further decreasing their prevalence over time [50].

To better demonstrate the effectiveness of PREDAC-FluB in simulating vaccine strain recommendations, we evaluated historical data on WHO-recommended vaccine strains. The results showed that the antigenic coverage of vaccine strains exhibited a distinct ‘inverted V-shaped’ distribution over time, with the highest coverage at the time of recommendation and a subsequent decline. Antigenic coverage, which reflects the antigenic similarity between vaccine strains and circulating strains, is an important indicator for predicting vaccine effectiveness. Therefore, we compared the limited vaccine effectiveness data (Supplementary Table S5) reported by the US CDC with the antigenic coverage of vaccine strains. The results indicated that for the Victoria lineage, when the antigenic coverage was below 30%, vaccine effectiveness was around 50%. However, during the 2017–2018 influenza season, vaccine effectiveness reached 76% despite a coverage rate below 20%. Additionally, in the 2023–2024 influenza season, vaccine effectiveness could reach 83.5% when the vaccine strain had high coverage (Supplementary Fig. S4a). For the Yamagata lineage, vaccine effectiveness generally correlated with high antigenic coverage, except in the 2012–2013 influenza season, when vaccine effectiveness reached 66% despite an antigenic coverage of less than 40% (Supplementary Fig. S4b). However, vaccine effectiveness can also be influenced by other factors, such as antigenic drift of the virus, mutations in vaccine production (e.g., egg adaptation), the immune status of the vaccinated population, and the immune response following vaccination [51–53]. Therefore, relying solely on antigenic coverage to assess vaccine effectiveness has limitations. In future studies, a comprehensive evaluation of influenza vaccine effectiveness should incorporate multidimensional data, including vaccine type, viral mutations, host health status, immune response, epidemiological data, and the specific needs of high-risk populations.

In conclusion, our study introduces PREDAC-FluB, a novel model that combines evolutionary information and physicochemical properties to predict antigenicity in influenza B viruses. The model’s robust performance in cross-validation and independent testing underscores its potential as a reliable tool for antigenicity prediction. By elucidating the evolutionary dynamics of influenza B viruses and their impact on antigenic drift, our work provides valuable insights for vaccine design and strain selection. As the first model of its kind, PREDAC-FluB paves the way for future research aimed at enhancing our understanding of influenza B antigenic evolution and improving vaccine efficacy. Additionally, with minor adjustments—such as fine-tuning the model to capture other influenza subtype- or pathogen-specific sequence characteristics—the framework of PREDAC-FluB could be readily adapted to other influenza subtypes or pathogens.

**Key Points**

- We fully leverage the advantages of convolutional neural networks (CNNs) to extract spatial feature representations through the receptive field of its convolutional layer and capture the complex dependencies between amino acid sites, thus laying a solid foundation for our PREDAC-FluB model, enabling it to effectively simulate the antigenic evolution of seasonal influenza B viruses.
- PREDAC-FluB specifically constructs a spatially oriented representation of the HA1 sequence for the convolutional architecture of the CNN model, which enables in-depth exploration of the interactions between amino acids in the HA1 sequence.
- With the help of the ESM2 pre-trained language model, PREDAC-FluB can effectively capture the deep dependencies between amino acid sites in the HA1 sequence, extract rich evolutionary information from large-scale sequence datasets, provide deep and rich representations for the sequence, and comprehensively evaluate the comprehensive contribution of point mutations in the HA1 sequence to antigenic variation.
- PREDAC-FluB can accurately identify the main antigenic clusters in seasonal influenza B viruses, providing strong support for antigenic monitoring and vaccine development of influenza viruses.

## Supplementary Material

Supplementary_materials_bbaf308

## Data Availability

Source data and code of PREDAC-FluB can be obtained by contacting the author.
