# Supplementary material for: PREDAC-FluB: predicting antigenic clusters of seasonal influenza B viruses with protein language model embedding based convolutional neural network
Source: Brief Bioinform. 2025 Jul 16;26(4):bbaf308. doi: 10.1093/bib/bbaf308 (PMC12264208; doi:10.1093/bib/bbaf308)
Supplement: Supplementary_materials_bbaf308 [file supplementary_materials_bbaf308.docx]

## Details of PREDAC-FluB

Figure 1 illustrates the workflow of PREDAC-FluB, which is divided into two modules: model construction and antigenic cluster inference. In the model construction module, data collection and processing are first performed. and key physicochemical properties of amino acids—including nonpolar (hydrophobic), polar (hydrophilic), sulfur-containing, and charged categories—are curated from the AAindex1 database[1]. Subsequently, feature extraction is carried out. PREDAC-FluB takes paired HA1 sequences of influenza B viruses from the Victoria or Yamagata lineages as input. For each pair of HA1 sequences (sequence 1 and sequence 2), PREDAC-FluB generates a specially designed input matrix that is suitable for convolution operations and employs a spatially oriented representation. The input matrix includes two types of features: 7-dimensional physicochemical encodings (six predefined AAindex1 descriptors with continuous positional encoding per residue) and (2) 320-dimensional semantic embeddings generated by the ESM-2 pre-trained model. Ultimately, these spatially structured representations of sequence pairs are concatenated and fed into a Convolutional Neural Network (CNN). The model's performance is evaluated using metrics such as AUC, Accuracy, Precision, Recall, and F1-score to select the best-performing model.

In the antigenic cluster inference module, PREDAC-FluB utilizes a trained CNN model to forecast the antigenic relationships between strains of influenza B viruses. Based on these predictions, an antigenic correlation matrix is constructed, and antigenic clusters are inferred using UMAP-assisted K-means clustering. Finally, the epidemic process of the antigenic clusters is simulated.

## Generation of input matrix

The input matrix for the paired HA1 sequences (sequence 1 and sequence 2) is a spatially oriented representation of the HA1 sequence. The influenza B/Victoria and B/Yamagata strains each have 346 columns representing amino acid sites within their HA1 sequences, with documented mutations that can affect the virus's antigenicity and vaccine efficacy. This matrix integrates two types of features: (1) Physicochemical Features: These include the encoding of six selected physicochemical properties of amino acids (accessible surface, charge, hydrophobicity, hyindex, polarity, and volume) [2, 3]and a continuous encoding based on a predefined feature dictionary[4] (as detailed in [Supplementary Tables S1](https://pmc.ncbi.nlm.nih.gov/articles/PMC10859661/#sup1) and [S2](https://pmc.ncbi.nlm.nih.gov/articles/PMC10859661/#sup1)), each amino acid has a feature dimension of 7.we applied z-score normalization to the physicochemical feature set to ensure zero mean and unit variance. (2) ESM-2 Embedding Features: These are the embedding vectors derived from the pre-trained protein language model ESM-2, based on the HA1 sequence. We input the HA1 sequences of influenza B/Victoria or B/Yamagata lineages into the ESM-2 (esm2_t6_8M_UR50D) model and extract the output from the last layer as the embedding vector, which has a dimension of 320, and we retain the original scaling of these vectors. This vector encodes the "semantic meaning" of each amino acid within the context of the HA1 sequence.

Finally, the feature matrix of each amino acid (dimension 7) and the embedding vector (dimension 320) were concatenated directly to form a feature representation with a dimension of 327. For paired HA1 sequences, these feature representations are concatenated to create an input matrix with a dimension of 654. This integrated representation, combining amino acid features, their physicochemical properties, and ESM-2 embeddings, is referred to as the "ESM2-7-features" encoding.

## Prediction by the CNN model

The input matrix for paired influenza B/Victoria or B/Yamagata lineage strains is a two-dimensional spatial representation of the HA1 sequence, with dimensions *L**654 (*L*=346). This complexity poses challenges for traditional machine learning algorithms, prompting us to use a Convolutional Neural Network (CNN) model, which excels at processing high-dimensional inputs[5-7].

As shown in Figure 1, the CNN architecture processes the input matrix using one-dimensional convolutional computations due to the absence of spatial representation in the HA1 sequence. To capture interactions between an amino acid site with its surrounding sites, we employ a kernel size of 3 and stride of 1. Maxpooling with a kernel size of 2 and stride of 2 is used for downsampling to extract significant features from small spatial regions. The classification layer then leverages these abstract feature representations to determine the antigenic relationship between paired strains of influenza B/Victoria and B/Yamagata lineages.

**References**

1. S. Kawashima, P. Pokarowski, M. Pokarowska, A. Kolinski, T. Katayama, M. Kanehisa. AAindex: amino acid index database, progress report 2008, Nucleic Acids Res 2008;36:D202-205.

2. J. L. Virelizier. Host defenses against influenza virus: the role of anti-hemagglutinin antibody, J Immunol 1975;115:434-439.

3. Velislava N Petrova, Colin A Russell. The evolution of seasonal influenza viruses, Nature Reviews Microbiology 2018;16:47-60.

4. M. W. Tenforde, R. J. G. Kondor, J. R. Chung, R. K. Zimmerman, M. P. Nowalk, M. L. Jackson, L. A. Jackson, A. S. Monto, E. T. Martin, E. A. Belongia, H. Q. McLean, M. Gaglani, A. Rao, S. S. Kim, T. J. Stark, J. R. Barnes, D. E. Wentworth, M. M. Patel, B. Flannery. Effect of Antigenic Drift on Influenza Vaccine Effectiveness in the United States-2019-2020, Clin Infect Dis 2021;73:e4244-e4250.

5. S. Min, B. Lee, S. Yoon. Deep learning in bioinformatics, Brief Bioinform 2017;18:851-869.

6. J Qiu, T Qiu, Y Yang, D Wu, Z Cao. Incorporating structure context of HA protein to improve antigenicity calculation for influenza virus A/H3N2, Sci Rep 2016;6:31156.

7. Z. Chen, X. Liu, P. Zhao, C. Li, Y. Wang, F. Li, T. Akutsu, C. Bain, R. B. Gasser, J. Li, Z. Yang, X. Gao, L. Kurgan, J. Song. iFeatureOmega: an integrative platform for engineering, visualization and analysis of features from molecular sequences, structural and ligand data sets, Nucleic Acids Res 2022;50:W434-W447.


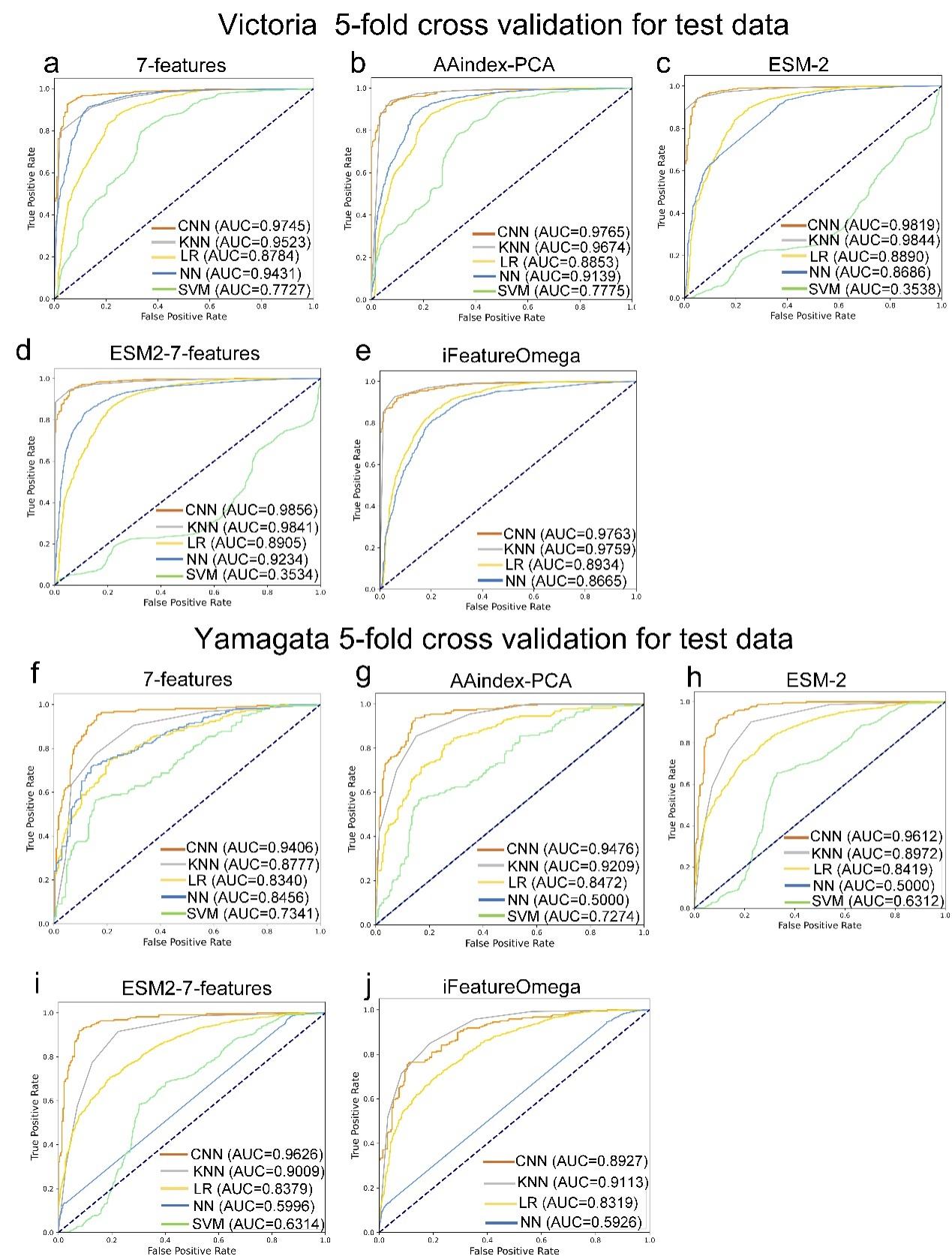
 **Figure S1**. ROC curves for 5-fold cross-validations of PREDAC-FluB and four machine learning models with five feature encodings in the independent test sets. ROC curves for feature encoding of 7-features (**a**), AAindex-PCA (**b**), ESM-2 embeddings (**c**), ESM2-7-features (**d**) and iFeatureOmega (**e**) on influenza B/Victoria viruses. ROC curves for feature encoding of 7-features (**f**), AAindex-PCA (**g**), ESM-2 embeddings (**h**), ESM2-7-features (**i**) and iFeatureOmega (**j**) on influenza B/Yamagata viruses.


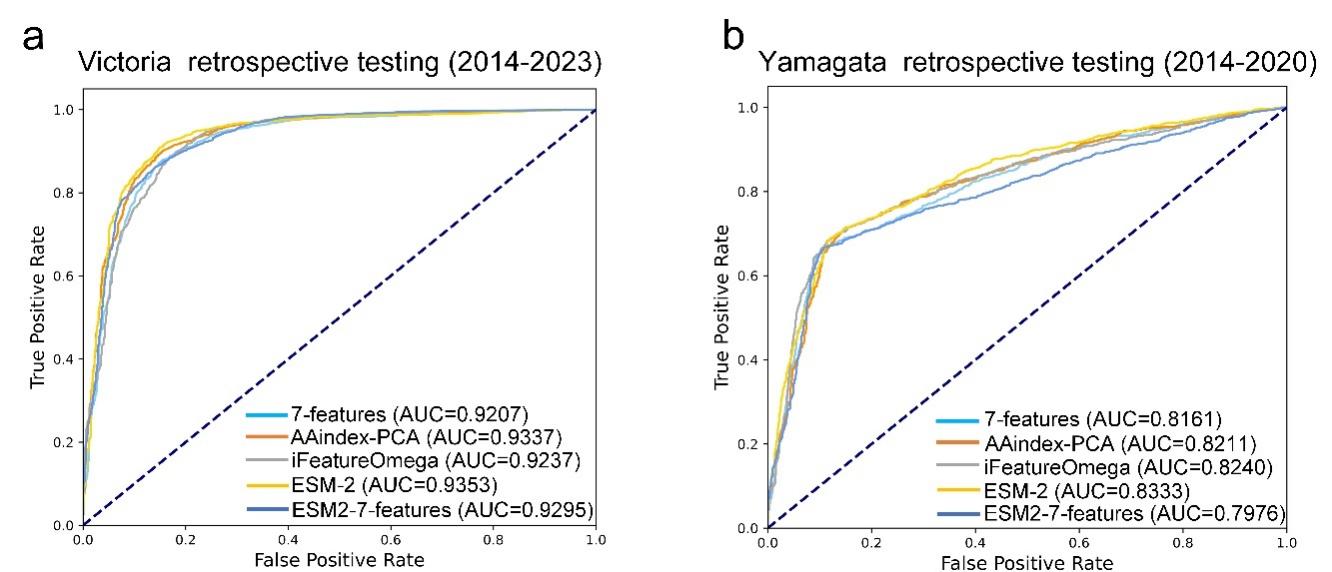
 **Figure S2.** Comparative Performance of ESM2-7-features Encoding vs. Other Feature Encodings with CNN Model in Retrospective Testing. **(a)** ROC curves for feature encodings of 7-features, AAindex-PCA, ESM-2 embeddings, ESM2-7-features, and iFeatureOmega on influenza B/Victoria viruses using the CNN model. **(b)** ROC curves for feature encodings of 7-features, AAindex-PCA, ESM-2 embeddings, ESM2-7-features, and iFeatureOmega on influenza B/Yamagata viruses using the CNN model.


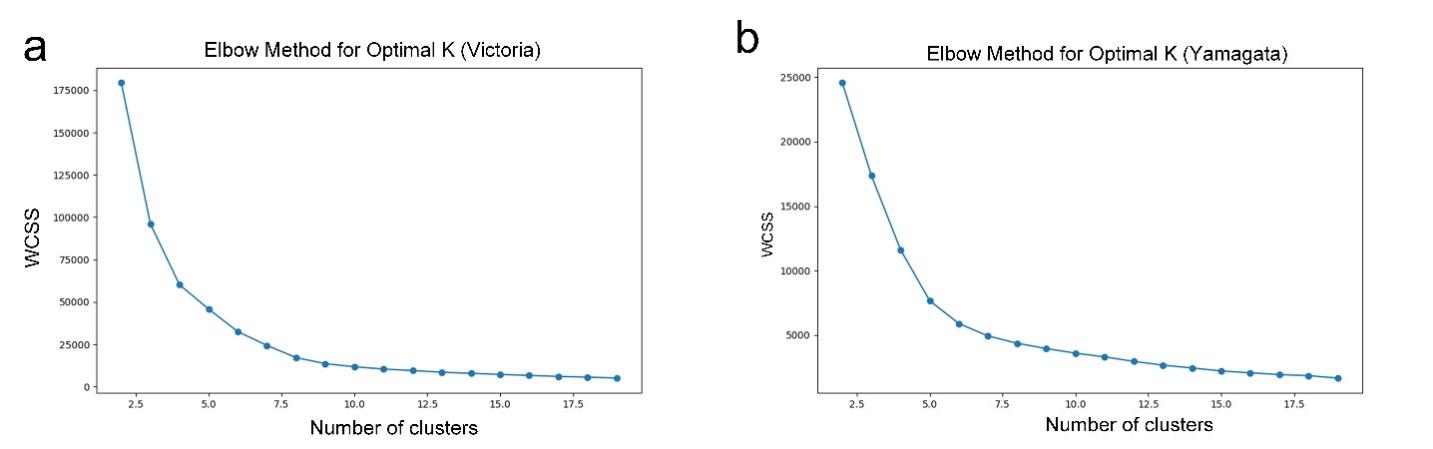


**Figure S3.** Optimal Cluster Determination for Influenza B Virus Lineages via the Elbow Method. (**a**) The Elbow Method is utilized to determine the optimal number of clusters (K) for the B/Victoria lineage, while (**b**) the same method is employed to establish the best cluster count (K) for the B/Yamagata lineage.


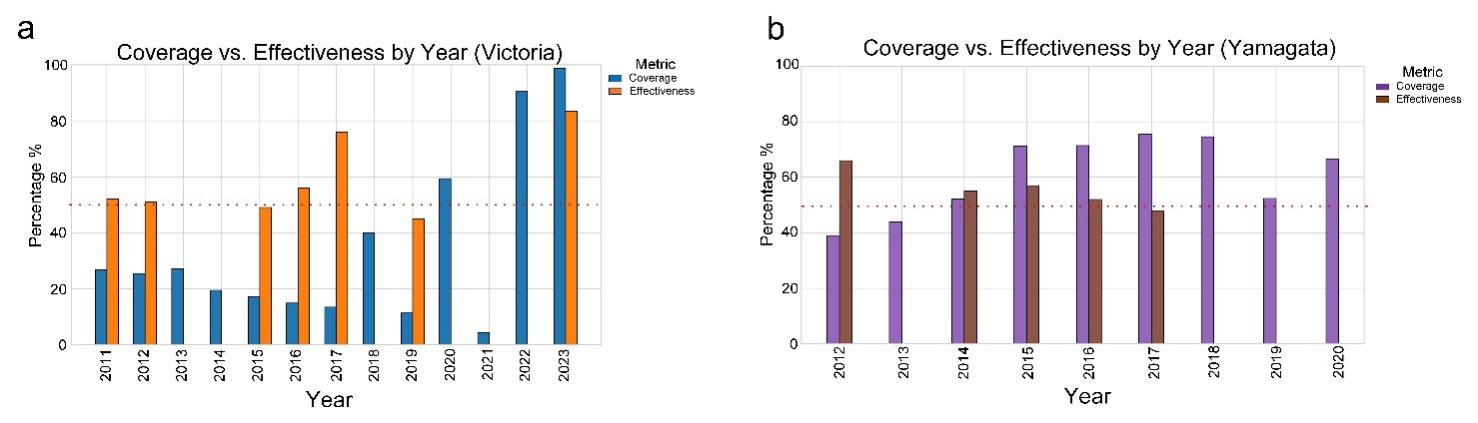


**Figure S4**. The coverage and effectiveness of Vaccine strains. (**a**) Annual Coverage vs. Effectiveness for Victoria lineage, blue bars represent antigen coverage, and orange bars show vaccine effectiveness for Victo ria lineage from 2011 to 2023. (**b**) Annual Coverage vs. Effectiveness for Yamagata lineage, purple bars represent antigen coverage, and brown bars show vaccine effectiveness for Yamagata lineage from 2012 to 2020. The red dashed line marks the 50% threshold of coverage and effectiveness.

**Supplementary Table S1.** Feature dictionary for influenza B/Victoria viruses.: six physicochemical features with corresponding optimal entries and continuous encodings for 20 amino acids.

|  | Continuous encoding | Accessible surface | Charge | Hydrophobicity | Hyindex | Polarity | Volume |
| --- | --- | --- | --- | --- | --- | --- | --- |
| A | 0 | 0.146567718 | 0 | 0.0125 | 0 | 0 | 0.169461078 |
| R | 0.05 | 0.614100186 | 1 | -0.177083333 | 0.25 | 1 | 0.725748503 |
| N | 0.1 | 0.249845393 | 0 | 0.052083333 | 1 | 0 | 0.389820359 |
| D | 0.15 | 0.215831787 | -1 | -0.041666667 | 1 | -1 | 0.304191617 |
| C | 0.2 | 0.429189858 | 0 | 0.102083333 | 0 | 0 | 0.313772455 |
| Q | 0.25 | 0.348175634 | 0 | 0.064583333 | 1 | 0 | 0.531137725 |
| E | 0.3 | 0.315398887 | -1 | -0.020833333 | 0.5 | -1 | 0.482035928 |
| G | 0.35 | 0 | 0 | 0.04375 | 0 | 0 | 0 |
| H | 0.4 | 0.574520717 | 0 | -0.466666667 | 0.5 | 0 | 0.554491018 |
| I | 0.45 | 0.58812616 | 0 | 0.725 | 0 | 0 | 0.649700599 |
| L | 0.5 | 0.62585034 | 0 | 0.729166667 | 0 | 0 | 0.649700599 |
| K | 0.55 | 0.325293754 | 1 | -0.3375 | 0.25 | 1 | 0.692215569 |
| M | 0.6 | 0.680272109 | 0 | 0.04375 | 0 | 0 | 0.611976048 |
| F | 0.65 | 0.811379097 | 0 | 1 | 0 | 0 | 0.77245509 |
| P | 0.7 | 0.185528757 | 0 | 0.147916667 | 0 | 0 | 0.37245509 |
| S | 0.75 | 0.140383426 | 0 | -0.129166667 | 1 | 0 | 0.171856287 |
| T | 0.8 | 0.269635127 | 0 | 0.135416667 | 1 | 0 | 0.348502994 |
| W | 0.85 | 1 | 0 | 0.477083333 | 0.25 | 0 | 1 |
| Y | 0.95 | 0.70995671 | 0 | 0.39375 | 1 | 0 | 0.796407186 |
| V | 1 | 0.482993197 | 0 | 0.33125 | 0 | 0 | 0.48742515 |
| - | 0.9 | 0.9 | 0.9 | 0.9 | 0.9 | 0.9 | 0.9 |

**Supplementary Table S2.** Feature dictionary for influenza B/Yamagata viruses: six physicochemical features with corresponding optimal entries and continuous encodings for 20 amino acids.

|  | Continuous encoding | Accessible surface | Charge | Hydrophobicity | Hyindex | Polarity | Volume |
| --- | --- | --- | --- | --- | --- | --- | --- |
| A | 0 | 0.140571429 | 0 | 0.230188679 | 0 | 0 | 0.14661215 |
| R | 0.05 | 0.905142857 | 1 | 0.226415094 | 0.25 | 1 | 0.792056075 |
| N | 0.1 | 0.509714286 | 0 | 0.022641509 | 1 | 0 | 0.401869159 |
| D | 0.15 | 0.515428571 | -1 | 0.173584906 | 1 | -1 | 0.339369159 |
| C | 0.2 | 0 | 0 | 0.403773585 | 0 | 0 | 0.299649533 |
| Q | 0.25 | 0.608 | 0 | 0 | 1 | 0 | 0.553154206 |
| E | 0.3 | 0.602285714 | -1 | 0.177358491 | 0.5 | -1 | 0.518107477 |
| G | 0.35 | 0.102857143 | 0 | 0.026415094 | 0 | 0 | 0 |
| H | 0.4 | 0.402285714 | 0 | 0.230188679 | 0.5 | 0 | 0.589369159 |
| I | 0.45 | 0.083428571 | 0 | 0.837735849 | 0 | 0 | 0.598130841 |
| L | 0.5 | 0.138285714 | 0 | 0.577358491 | 0 | 0 | 0.592873832 |
| K | 0.55 | 1 | 1 | 0.433962264 | 0.25 | 1 | 0.612733645 |
| M | 0.6 | 0.205714286 | 0 | 0.445283019 | 0 | 0 | 0.609813084 |
| F | 0.65 | 0.114285714 | 0 | 0.762264151 | 0 | 0 | 0.800233645 |
| P | 0.7 | 0.411428571 | 0 | 0.735849057 | 0 | 0 | 0.367406542 |
| S | 0.75 | 0.302857143 | 0 | 0.018867925 | 1 | 0 | 0.191004673 |
| T | 0.8 | 0.337142857 | 0 | 0.018867925 | 1 | 0 | 0.325350467 |
| W | 0.85 | 0.219428571 | 0 | 1 | 0.25 | 0 | 1 |
| Y | 0.95 | 0.453714286 | 0 | 0.709433962 | 1 | 0 | 0.801401869 |
| V | 1 | 0.093714286 | 0 | 0.498113208 | 0 | 0 | 0.439836449 |
| - | 0.9 | 0.9 | 0.9 | 0.9 | 0.9 | 0.9 | 0.9 |

**Supplementary Tables S3.** The performance of the model on the test set in the retrospective test for B/Victoria viruses.

| year | TP | FP | TN | FN | Accuracy | F1-score | Precision | Recall | AUC |
| --- | --- | --- | --- | --- | --- | --- | --- | --- | --- |
| 2014 | 201 | 23 | 44 | 20 | 0.85 | 0.90 | 0.90 | 0.91 | 0.88 |
| 2015 | 166 | 24 | 66 | 18 | 0.85 | 0.89 | 0.87 | 0.90 | 0.90 |
| 2016 | 625 | 51 | 66 | 29 | 0.90 | 0.94 | 0.92 | 0.96 | 0.92 |
| 2017 | 512 | 33 | 69 | 16 | 0.92 | 0.95 | 0.94 | 0.97 | 0.93 |
| 2018 | 618 | 46 | 37 | 8 | 0.92 | 0.96 | 0.93 | 0.99 | 0.83 |
| 2019 | 1253 | 76 | 101 | 41 | 0.92 | 0.96 | 0.94 | 0.97 | 0.93 |
| 2020 | 663 | 16 | 81 | 18 | 0.96 | 0.98 | 0.98 | 0.97 | 0.98 |
| 2021 | 431 | 36 | 129 | 50 | 0.87 | 0.91 | 0.92 | 0.90 | 0.89 |
| 2022 | 781 | 54 | 119 | 9 | 0.93 | 0.96 | 0.94 | 0.99 | 0.93 |
| 2023 | 518 | 59 | 171 | 8 | 0.91 | 0.94 | 0.90 | 0.98 | 0.97 |

**Supplementary Tables S4.** The performance of the model on the test set in the retrospective test for B/Yamagata viruses.

| year | TP | FP | TN | FN | Accuracy | F1-score | Precision | Recall | AUC |
| --- | --- | --- | --- | --- | --- | --- | --- | --- | --- |
| 2014 | 193 | 39 | 196 | 66 | 0.79 | 0.79 | 0.83 | 0.75 | 0.80 |
| 2015 | 156 | 71 | 244 | 81 | 0.72 | 0.67 | 0.69 | 0.66 | 0.78 |
| 2016 | 116 | 8 | 120 | 100 | 0.69 | 0.68 | 0.94 | 0.54 | 0.85 |
| 2017 | 298 | 19 | 391 | 156 | 0.80 | 0.77 | 0.94 | 0.66 | 0.83 |
| 2018 | 209 | 67 | 421 | 85 | 0.81 | 0.73 | 0.76 | 0.71 | 0.85 |
| 2019 | 60 | 14 | 50 | 11 | 0.81 | 0.83 | 0.81 | 0.85 | 0.83 |
| 2020 | 2 | 5 | 4 | 0 | 0.55 | 0.44 | 0.29 | 1.00 | 1.00 |

**Supplementary Tables S5.** The data of vaccine effectiveness for the corresponding influenza season reported by the US CDC for the Victoria lineage and the Yamagata lineage viruses, respectively.

| Flu season | Victoria | Yamagata | Link | |
| --- | --- | --- | --- | --- |
| 2011-2012 | 52 | 66 | <https://www.cdc.gov/flu/vaccines-work/2011-2012.html> |  |
| 2012-2013 | 51 | 66 | <https://pmc.ncbi.nlm.nih.gov/articles/PMC7643742/#CIT0005> | |
| 2013-2014 | NA | NA | <https://www.cdc.gov/flu/vaccines-work/2011-2012.html> | |
| 2014-2015 | NA | 55 | https://archive.cdc.gov/#/details?url=https://www.cdc.gov/flu/vaccines-work/2014-2015.html | |
| 2015-2016 | 49 | 57 | <https://archive.cdc.gov/#/details?url=https://www.cdc.gov/flu/vaccines-work/2015-2016.html> | |
| 2016-2017 | 56 | 52 | <https://archive.cdc.gov/#/details?url=https://www.cdc.gov/flu/vaccines-work/2016-2017.html> | |
| 2017-2018 | 76 | 48 | https://archive.cdc.gov/#/details?url=https://www.cdc.gov/flu/vaccines-work/2017-2018.html | |
| 2018-2019 | NA | NA | https://archive.cdc.gov/#/details?url=https://www.cdc.gov/flu/vaccines-work/2018-2019.html | |
| 2019-2020 | 45 | NA | https://www.cdc.gov/flu-vaccines-work/php/effectiveness-studies/2019-2020.html | |
| 2020-2021 | NA | NA | NA | |
| 2021-2022 | NA |  | https://www.cdc.gov/flu-vaccines-work/php/effectiveness-studies/2021-2022.html | |
| 2022-2023 | NA |  | https://www.cdc.gov/flu-vaccines-work/php/effectiveness-studies/2022-2023.html | |
| 2023-2024 | 83.5# |  | https://www.cdc.gov/flu-vaccines-work/php/effectiveness-studies/2023-2024.html | |
| #The average value of children and adults, the effectiveness of the influenza B vaccine was 89% in children and 78% in adults. | | | | |
